# Epidemiology of out-of-hospital cardiac arrest in Scotland; impact of Scottish strategy and COVID-19 pandemic.

**DOI:** 10.23889/ijpds.v7i3.2045

**Published:** 2022-08-25

**Authors:** Nynke Halbesma, Andy Kent, Gareth Clegg

**Affiliations:** 1 University of Edinburgh; 2 Scottish Ambulance Services

## Objectives

Low Scottish survival rates after out-of-hospital cardiac arrest (OHCA) resulted the launch of the Scottish out-of-hospital cardiac arrest strategy in 2015. Data-linkage including SAS data and other datasets has enabled monitoring the impact of the strategy and COVID-19 on bystander cardiopulmonary resuscitation (bCPR) and 30 days survival rates.

## Approach

All incident OHCA cases between 2011 and 2020 cases where resuscitation has been attempted were identified in the SAS data. These OHCA cases have been linked with a broad range of other administrative datasets including unscheduled care datamart (UCD), hospitalisations (SMR01) and deaths data. The Community Health Index (CHI), a unique identifier used across all health records in Scotland enabled this data linkage through direct and probabilistic linkage (CHI retrieved and used for further linkage). Descriptive analysis and logistic regression analyses were used to assess the impact of both the implementation of the strategy and the COVID-19 pandemic.

## Results

Over 27,000 non-traumatic OHCAs with resuscitation attempted by the SAS, between 2011 and 2020 were identified. Successful data-linkage improved over time reaching >85% at the end of data collection. Confounder adjusted logistic regression showed higher adjusted odds of bCPR (OR=2.7) and 30days survival rates (7.3% versus 10.2% or increase from 44.5 per million population to 58.5 per million population) comparing 2014-15 and 2018-19 data. Initial analyses show that during the COVID-19 pandemic (March 2020-February 2021) bCPR percentages remained stable however 30days survival rates dropped to 6.9%.

## Conclusion

The launch of the Scottish OHCA strategy and subsequent initiatives to improve bCPR rates has led to an increase in bCPR and 30 days survival rates. However, the COVID-19 pandemic has had a negative impact on survival. Analyses are underway to gain insight in detailed explanations for these findings.

